# Methods to estimate breeding values in honey bees

**DOI:** 10.1186/s12711-014-0053-9

**Published:** 2014-09-19

**Authors:** Evert W Brascamp, Piter Bijma

**Affiliations:** Animal Breeding and Genomics Centre, Wageningen University, PO Box 338, 6700 AH Wageningen, The Netherlands

## Abstract

**Background:**

Efficient methodologies based on animal models are widely used to estimate breeding values in farm animals. These methods are not applicable in honey bees because of their mode of reproduction. Observations are recorded on colonies, which consist of a single queen and thousands of workers that descended from the queen mated to 10 to 20 drones. Drones are haploid and sperms are copies of a drone’s genotype. As a consequence, Mendelian sampling terms of full-sibs are correlated, such that the covariance matrix of Mendelian sampling terms is not diagonal.

**Results:**

In this paper, we show how the numerator relationship matrix and its inverse can be obtained for honey bee populations. We present algorithms to derive the covariance matrix of Mendelian sampling terms that accounts for correlated terms. The resulting matrix is a block-diagonal matrix, with a small block for each full-sib family, and is easy to invert numerically. The method allows incorporating the within-colony distribution of progeny from drone-producing queens and drones, such that estimates of breeding values weigh information from relatives appropriately. Simulation shows that the resulting estimated breeding values are unbiased predictors of true breeding values. Benefits for response to selection, compared to an existing approximate method, appear to be limited (~5%). Benefits may however be greater when estimating genetic parameters.

**Conclusions:**

This work shows how the relationship matrix and its inverse can be developed for honey bee populations, and used to estimate breeding values and variance components.

## Background

Currently, honey bees (*Apis mellifera*) draw a lot of public and scientific attention because of increased colony losses [1,2], which are partly caused by infection with Varroa mites [3]. Although selection is a promising way to improve Varroa tolerance of honey bees, estimation of breeding values is not common practice in this species [3,4]. One reason is that it requires an organised collection of data on a relevant scale, which is rarely the case in honey bees. Currently, estimation of breeding values in honey bees is performed only in the German Beebreed program (http://www.beebreed.eu), for which breeding values are estimated from data that are collected annually on about 6000 colonies [4]. For specifics on the genetic evaluation method used in the Beebreed program that we refer to as BER for Bienefeld, Ehrhardt and Reinhardt, please see [5].

Methodology for breeding value estimation in honey bees has drawn the attention of animal breeders [6–8]. They discussed the calculation of additive genetic relationships that account for the fact that the workers in a colony descend from a single diploid queen and 10 to 20 haploid drones. One approach that focused on the haplo-diploid nature of honey bees [6,7] suggested that an allelic relationship matrix that contains relationships between gametes instead of between individuals, can be adapted to the specifics of honey bee ancestry. Another approach focused on the uncertainty about the father of an individual [8] and suggested that methods developed for the use of mixed semen of sires can be adapted to honey bees. To our knowledge, these approaches have not been developed for implementation.

Breeding value estimation with an animal model builds on the work of Henderson [9], who derived the required inverse of the numerator relationship matrix using a decomposition of breeding values into Mendelian sampling terms. Because Mendelian sampling terms are mutually independent, the covariance matrix of these terms is diagonal, which facilitates inversion. However, it is not a diagonal matrix in honey bees [5], because the paternal contribution to the additive genetic relationship differs between workers in the same colony and workers in different colonies. Bienefeld et al. [5] solved this problem by using an approximation, in which both contributions are averaged and breeding values are estimated with an animal model. As a result, the matrix of Mendelian sampling terms is diagonal again, but the weighting of information of relatives is approximate.

The purpose of this paper was to develop a method, referred to as BB (for Brascamp and Bijma), to calculate the relationship matrix and its inverse for honey bee populations, in order to estimate breeding values and genetic parameters with an animal model. We used the approach of Henderson [9] as a starting point to derive the required procedures, taking in account the biology of the reproduction in honey bees. We also summarize the BER method and provide insight into the quantitative differences between the BB and BER methods, using Monte Carlo simulation in a simple example.

### Reproduction of honey bees and colony observations

There are three types of individuals in honey bees: queens, workers and drones. Queens and workers are diploid, while drones are haploid. A colony of honey bees consists of a single fertilized queen, around ten thousand workers and several hundred drones. Workers contribute, for example, to the collection of pollen and nectar, the production of wax and nursing of the queen, but have no role in reproduction. Drones, in contrast, only serve for reproduction.

The description of the reproduction cycle in honey bees starts with a virgin queen. Soon after emerging from the brood cell, the virgin queen leaves the colony (nuptial flight) to mate in flight with multiple drones that come essentially from other colonies. These drones concentrate in so-called drone congregation areas, bringing together queens and drones in a range as large as 10 km. Drones die immediately after mating, which means that they can mate to a single queen only. Queens are mated only during their nuptial flight, or perhaps during a few nuptial flights within a small time slot and they cannot be mated again later in life. The queen stores a life lasting stock of millions of sperm cells in her spermatheca. After returning to their colony, mated queens produce two types of eggs, fertilized and unfertilized eggs. Fertilized eggs usually develop into diploid workers, while unfertilized eggs develop into haploid drones. Occasionally, an offspring of a fertilized egg receives a special diet from the workers and as a consequence develops into a virgin queen, which means that both workers and a virgin queen develop from a fertilized egg. The haploid drones that develop from unfertilized eggs have no father. They can be considered as flying gametes, and produce cloned sperm (*i.e.*, all gametes produced by a drone are genetically identical).

Controlled mating of queens requires control of drones, which is possible only by restricting the presence of drone-producing queens with a particular pedigree on isolated mating stations (*e.g.* islands), or by artificial insemination. Under normal circumstances, in a colony, drones are produced along with workers, but the production of drones can be stimulated by management measures. Note that queens are always mated to multiple drones, both with natural mating and artificial insemination. Thus, the worker progeny of a queen descend from multiple drones. This situation resembles that with mixed semen in the case of *e.g.* pigs, for which the progeny of a sow derive from multiple boars. With respect to genetic relationships, the key difference between bees and mixed semen in pigs is that each piglet descends from a genetically unique paternal gamete, while subsets of the workers in a colony descend from the same drone and therefore from genetically identical paternal gametes.

**Figure 1:**
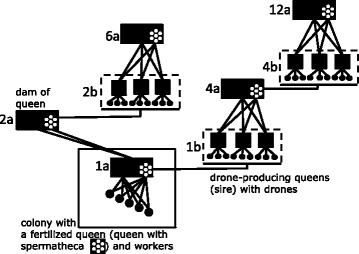
**The Beebreed breeding system.** Rectangles indicate fertilized queens with sperm cells in their spermatheca. The sperm cells derive from drones (small circles) produced by drone-producing queens (squares). The picture illustrates the pedigree of queen 1a in a colony with workers that derive from the queen and the sperm cells in the spermatheca. The numbers 1a-12a given to queens and 1b-4b given to drone-producing queens are those used in the Beebreed system.

The Beebreed system is shown in Figure 1 (see reference [10]). On the maternal side, the pedigree is straightforward because each queen (*e.g.*, 1a) has a single queen as mother (2a) but the paternal (*i.e.* drone) side is more complex. A queen is mated to multiple drones that descend from a group of drone-producing queens (1b). These drone-producing queens descend from a single mother (4a), which, in turn, has also been mated to drones that descend from a group of drone-producing queens (4b) with a single mother (12a). Note that, although drone-producing queens are also mated, the drones they produce contain genes of the queen only i.e. not of its mate.

Drones cannot be not traced and it is unknown how many and which drones have mated to the queen. As a consequence, the contribution of each drone-producing queen to the offspring of the queen is unknown. For this reason, the group of drone-producing queens can be treated as a single “individual”, which we will refer to as the sire of the workers of the colony. In Figure 1, for example, the three drone-producing queens in 1b together constitute the sire of the workers in the colony of queen 1a. By grouping the drone-producing queens into a single sire, each individual in the pedigree has precisely two parents, a queen and a sire. This grouping makes it easier to trace the pedigree without loss of information.

Observations in honey bees are on colony performance, and may relate to traits like honey production, behaviour and disease resistance [4]. The performance of a colony is affected by the joint genetic effects of the ten thousand workers (called worker effect) and by the genetic effects of the queen (called queen effect). Colony performance results from the action of the workers and the interaction between workers, but also from the effects of the queen on the workers, for example, due to the number of workers produced or by producing pheromones that affect worker behaviour. However, workers also affect the behaviour of the queen. Despite these different interactions, the performance of a colony can be partitioned into an additive worker effect and an additive queen effect, based on the principle of least squares [11]. Conceptually, this is similar to defining the average effect of an allele for a locus showing dominance, and to maternal effects in mammals [12]. Several studies [10,13] have shown that the contribution of queen effects to colony performance is considerable, although smaller than that of worker effects, while the genetic correlation between worker and queen effects is negative.

## Methods

In the following paragraphs, we consider three types of individuals: (i) queens, (ii) sires, and (iii) groups of workers in a colony, referred to as worker groups. Queens are single individuals, while sires and worker groups are aggregates of individuals. With this categorisation, we cover individuals responsible for the phenotypes (queens and worker groups) and individuals in the pedigree (queens and sires). To emphasize that worker groups and sires consist of groups of individuals, we will write their breeding values as averages, using $\overline{A}$, while using *A* for the breeding value of a queen. Since breeding values are to be estimated on all three categories, the size of the numerator relationship matrix will be twice the number of queens (because each colony has one queen and one worker group) plus the number of sires.

The performance of a colony, *P*_*c*_, can be written as the sum of a worker effect, ${\overline{A}}_w^W$, a queen effect, ${A}_d^Q$, and a non-heritable residual, *E*_*c*_,:

1 $$ {P}_c={\overline{A}}_w^W+{A}_d^Q+{E}_c, $$

where ${\overline{A}}_w^W$ is the average breeding value of the worker group for worker effect, and ${A}_d^Q$ the breeding value of the dam of workers, *i.e.*, the queen in the colony, for queen effect. Thus, superscript *W* denotes the worker effect, superscript *Q* the queen effect, and subscript *c* denotes a colony, *w* the worker group of the colony, and *d* the queen of the colony. Equation (1) shows that the expected colony performance is equal to the sum of the queen effect and the worker effect.

Candidates for selection are the queens of the colonies, either to produce the next generation of queens, or to produce the next generation of sires. It is important to realize that the queens were mated early in life and cannot be re-mated, which means that selection focuses on the combination of a queen and the drones it was mated to. This situation clearly differs from the usual situation in animal breeding, where parents of both sexes are selected separately and mated afterwards. Thus, when selecting queens, the criterion of interest is the estimated breeding value of an average female offspring of a mated queen, say *i*, which equals the estimated breeding value of the workers in the queen’s colony:

2 $$ {\widehat{A}}_i^W+{\widehat{A}}_i^Q={\widehat{A}}_w^W+{\widehat{A}}_w^Q. $$

### Mixed model

Here, we consider a single trait situation, where each observation is affected by the worker effect of the worker group in the colony and the queen effect of the queen in the colony. Thus, observations on colonies are modelled as:

3 $$ \mathbf{y}=\mathbf{Xb}+{\mathbf{Z}}_{\mathbf{W}}{\mathbf{a}}_{\mathbf{W}}+{\mathbf{Z}}_{\mathbf{Q}}{\mathbf{a}}_{\mathbf{Q}}+\mathbf{e}, $$

where **y** is the vector of observations on colonies, **b** a vector of fixed effects with incidence matrix **X**, **a**_**W**_ a vector of worker effects with incidence matrix **Z**_**W**_, **a**_**Q**_ a vector of queen effects with incidence matrix **Z**_**Q**_, and **e** a vector of residuals. In both methods BB and BER, **Z**_**W**_ and **Z**_**Q**_ simply contain 1 s to connect the breeding value to the observation. In Equation (3), the residual includes the non-genetic effects due to both the queen and its workers. However, since a queen has only one colony throughout its life and workers contribute to a single colony only, those two non-genetic effects can be combined into a single residual that is independent between colonies: $var\left(\mathbf{e}\right)=\mathbf{I}{\sigma}_e^2$. Estimates of the fixed effects and breeding values are obtained by solving the following mixed model equations [14]:

4 $$ \begin{array}{l}\left[\begin{array}{ccc}\hfill {\mathbf{X}}^{\mathbf{\prime}}\mathbf{X}\hfill & \hfill {\mathbf{X}}^{\mathbf{\prime}}{\mathbf{Z}}_{\mathbf{W}}\hfill & \hfill {\mathbf{X}}^{\mathbf{\prime}}{\mathbf{Z}}_{\mathbf{Q}}\hfill \\ {}\hfill {\mathbf{Z}}_{\mathbf{W}}^{\mathbf{\prime}}\mathbf{X}\hfill & \hfill {\mathbf{Z}}_{\mathbf{W}}^{\mathbf{\prime}}{\mathbf{Z}}_{\mathbf{W}}+{\mathbf{A}}^{-1}{\upalpha}_1\hfill & \hfill {\mathbf{Z}}_{\mathbf{W}}^{\mathbf{\prime}}{\mathbf{Z}}_{\mathbf{Q}}+{\mathbf{A}}^{-1}{\upalpha}_2\hfill \\ {}\hfill {\mathbf{Z}}_{\mathbf{Q}}^{\mathbf{\prime}}\mathbf{X}\hfill & \hfill {\mathbf{Z}}_{\boldsymbol{Q}}^{\mathbf{\prime}}{\mathbf{Z}}_{\mathbf{W}}+{\mathbf{A}}^{-1}{\upalpha}_2\hfill & \hfill {\mathbf{Z}}_{\mathbf{Q}}^{\mathbf{\prime}}{\mathbf{Z}}_{\mathbf{Q}}+{\mathbf{A}}^{-1}{\upalpha}_3\hfill \end{array}\right]\\ {}\left[\begin{array}{c}\hfill \boldsymbol{\upmu} \hfill \\ {}\hfill {\mathbf{a}}_{\mathbf{W}}\hfill \\ {}\hfill {\mathbf{a}}_{\mathbf{Q}}\hfill \end{array}\right]=\left[\begin{array}{c}\hfill {\mathbf{X}}^{\mathbf{\prime}}\mathbf{y}\hfill \\ {}\hfill {\mathbf{Z}}_{\mathbf{W}}^{\mathbf{\prime}}\mathbf{y}\hfill \\ {}\hfill {\mathbf{Z}}_{\mathbf{Q}}^{\mathbf{\prime}}\mathbf{y}\hfill \end{array}\right],\end{array} $$

Where **A** is the numerator relationships matrix and

5 $$ \begin{array}{l}\left[\begin{array}{cc}\hfill {\alpha}_1\hfill & \hfill {\alpha}_2\hfill \\ {}\hfill {\alpha}_2\hfill & \hfill {\alpha}_3\hfill \end{array}\right]=\\ {}{\left[\begin{array}{cc}\hfill {\sigma}_{A_W}^2\hfill & \hfill {r}_G{\sigma}_{A_W}{\sigma}_{A_Q}\hfill \\ {}\hfill {r}_G{\sigma}_{A_W}{\sigma}_{A_Q}\hfill & \hfill {\sigma}_{A_Q}^2\hfill \end{array}\right]}^{-1}{\sigma}_e^2.\ \end{array} $$

Here ${\sigma}_{A_W}^2$ and ${\sigma}_{A_Q}^2$ are the additive genetic variances for worker and queen effect, respectively, and *r*_*G*_ is the genetic correlation between these effects.

In the next section, we develop the method to derive **A**^− 1^ that is needed in Equation (4).

### Numerator relationship matrix

Henderson [9] derived a simple method to compute the inverse of a numerator relationship matrix. Consider the breeding value *A*_*i*_ of individual *i*, which is the sum of half the breeding value of its father, *A*_*s*_, half the breeding value of its mother, *A*_*d*_, and a Mendelian sampling term, *δ*_*i*_,

6 $$ {A}_i=\frac{1}{2}{A}_d+\frac{1}{2}{A}_s+{\delta}_i. $$

In matrix notation, the breeding values of all individuals in the pedigree may be represented by a vector **a**, such that:

7 $$ \mathbf{a}=\mathbf{Ma}+\mathbf{d}, $$

where **M** is a matrix connecting an individual to its parents, with offspring on the rows and parents on the columns. The row for an offspring contains two ½’s when both parents are known, one ½ when only one parent is known, and all 0’s when no parents are known. The vector **d** contains the Mendelian sampling terms. Let **A** denote the covariance matrix of **a**, the numerator relationship matrix, and **D** the covariance matrix of **d**. Under normal diploid inheritance, which is the most common in animal breeding, **D** is a diagonal matrix because the Mendelian sampling terms for different individuals are independent of each other. From Equation (7), the vector of Mendelian sampling terms can be written as **d** = (**I** − **M**)**a**. It follows that **a** = (**I** − **M**)^− 1^**d** and consequently **A** = **var**((**I** − **M**)^− 1^**d**) = (**I** − **M**)^− 1^**D**(**I** − **M**)^'− 1^. Taking the inverse yields:

8 $$ {\mathbf{A}}^{-1}={\left(\mathbf{I}-\mathbf{M}\right)}^{\prime }{\mathbf{D}}^{-1}\left(\mathbf{I}-\mathbf{M}\right). $$

Equation (8) is used as the basis for a simple method to invert **A** [15], because **I** and **M** are simple matrices and **D** is a diagonal matrix for most livestock species.

Equations (6) through (8) hold for honey bees as well, but **D** is no longer a diagonal matrix. In the following, we derive the diagonals and off-diagonals of **D**, considering the three types of individuals defined above: queens, sires and worker groups. Because **D** is the same for all traits of interest, we do not distinguish between worker and queen effects, and therefore drop the *W* and *Q* superscripts.

### Diagonal elements of **D**

#### Queens

The breeding value of a queen, say *i*, can be decomposed into parental terms and a Mendelian sampling deviation:

9 $$ {A}_i=\frac{1}{2}{A}_d+\frac{1}{2}{\overline{A}}_s+{\delta}_i. $$

The interesting feature is in the diagonal element of **D** for queens, which is given by:

10 $$ {\mathbf{D}}_{ii}=\frac{var\left({\delta}_i\right)}{\sigma_A^2}, $$

where ${\sigma}_A^2$ is the additive genetic variance in the base population. The *var*(*δ*_*i*_) follows from writing the variance of Equation (9) and solving the resulting expression for *var*(*δ*_*i*_). Taking the variance of Equation (9) yields:

11 $$ \begin{array}{l}\mathrm{var}\left({A}_i\right)={\sigma}_A^2\left(1+{F}_i\right)=\frac{1}{4}{\sigma}_A^2\left(1+{F}_d\right)+\\ {}\frac{1}{4} var\left({\overline{A}}_s\right)+\frac{1}{2} cov\left({A}_d,{\overline{A}}_s\right)+ var\left({\delta}_i\right),\end{array} $$

where *F* denotes the coefficient of inbreeding. Note that var(*A*_*i*_) denotes the variance of the breeding value for the individual of interest, whereas ${\sigma}_A^2$ in Equation (10) denotes additive genetic variance in the base population. The variance of the breeding value of the sire in Equation (11) is given by:

12 $$ \mathrm{var}\left({\overline{A}}_s\right)=\frac{\sigma_A^2}{S}\left[\left(1+{F}_s\right)+\left(S-1\right){a}_{ss}\right], $$

where *S* is the number of drone-producing queens constituting a sire, *F*_*s*_ the inbreeding coefficient of the drone-producing queens, and *a*_*ss*_ the additive genetic relationship between those drone-producing queens. Because all drone-producing queens within a sire have the same pedigree (Figure 1), they all have the same value for *F*_*s*_ and *a*_*ss*_. Furthermore, $\frac{1}{2} cov\left({A}_d,{\overline{A}}_s\right)={F}_i{\sigma}_A^2$, so that *F*_*i*_ cancels from Equation (11). Finally, solving this equation for *var*(*δ*_*i*_) yields (Appendix 1):

13 $$ \begin{array}{l} var\left({\delta}_i\right)=\frac{1}{4}{\sigma}_A^2\left(1-{F}_d\right)+\frac{1}{4}{\sigma}_A^2\left(1-{F}_s\right)+\\ {}\frac{1}{4}{\sigma}_A^2\frac{\left(S-1\right)}{S}\left(1+{F}_s-{a}_{ss}\right).\end{array} $$

The first component in Equation (13) represents the variance due to the Mendelian sampling of maternal gametes, the second one the variance due to the Mendelian sampling of gametes of an individual drone-producing queen, and the third one the variance due to the sampling among drone-producing queens. Note that this equation can be applied when both parents of individual *i* are known. If this is not the case, refer to Appendix 1.

#### Sires

The breeding value of a sire can be decomposed into parental terms and a sampling deviation:

14 $$ {\overline{A}}_i=\frac{1}{2}{A}_d+\frac{1}{2}{\overline{A}}_s+{\overline{\delta}}_i. $$

Since a sire is a group of *S* (drone-producing) queens, the ${\overline{\delta}}_i$ in Equation (14) is the average of *S* individual δ values as defined by Equation (9):

15 $$ {\overline{\delta}}_i=\frac{1}{S}{\displaystyle \sum_{j=1}^S{\delta}_{ij}}. $$

Taking the variance of Equation (15) shows that the sampling variance for sires equals:

16 $$ var\left({\overline{\delta}}_i\right)=\frac{var\left({\delta}_i\right)}{S}+\frac{S-1}{S} cov\left({\delta}_{ij},{\delta}_{ik}\right), $$

where *var*(*δ*_*i*_) is given by Equation (13). (Since all *δ*_*ij*_ have the same variance, we dropped subscript *j* in *var*(*δ*_*i*_)).

Usually, in animal breeding, Mendelian sampling terms of individuals are independent because each individual descends from unique gametes, so that *cov*(*δ*_*ij*_, *δ*_*ik*_) = 0. For example, in pigs for which mixed semen is used, two offspring born from the same artificial insemination of a sow have independent Mendelian sampling terms because they derive from different gametes. In that case, the covariance between sibs is completely taken care of by the pedigree, as described by the term **Ma** in Equation (7), so that *cov*(*δ*_*ij*_, *δ*_*ik*_) = 0. The situation is different in the honey bee, because a drone produces clonal sperm that consists of identical gametes. As a consequence, two offspring of the same drone derive from identical paternal gametes, and therefore have identical paternal Mendelian sampling terms. Offspring can descend from the same drone if and only if they have the same mother, because drones can mate only once. Since drone-producing queens within a sire have the same mother, they may descend from the same drone. Thus, the paternal covariance between two drone-producing queens within the same sire, say *j* and *k*, arises not only because they share a common sire (a drone-producing queen), but also because they may descend from the same drone. The size of the covariance between the Mendelian sampling terms of two offspring of the same queen and sire combination, which can be written as *cov*(*δ*_*ij*_, *δ*_*ik*_) = *cov*(*δ*_*FS*_), where subscript *FS* denotes full-sibs, is discussed in the next paragraph. Here we only rewrite Equation (16) to become:

17 $$ var\left({\overline{\delta}}_i\right)=\frac{var\left({\delta}_i\right)}{S}+\frac{S-1}{S}\  cov\left({\delta}_{FS}\right), $$

where *var*(*δ*_*i*_) is given by Equation (13). The diagonal elements for sires are equal to:

18 $$ {\mathbf{D}}_{ii}=\frac{var\left({\overline{\delta}}_i\right)}{\sigma_A^2}. $$

#### Worker groups

Since worker groups and sires are groups of individuals that descend from a single mother, the decomposition of the breeding value of a worker group is the same as for a sire (Equation (14)). Analogous to Equation (17), the variance of the average sampling deviation of the ten thousand workers in a colony can be written as $var\left({\overline{\delta}}_i\right)=\frac{var\left({\delta}_i\right)}{n}+\frac{n-1}{n} cov\left({\delta}_{FS}\right)$, *n* denoting the number of workers in a colony. Since *n* is very large, it follows that:

19 $$ var\left({\overline{\delta}}_i\right)= cov\left({\delta}_{FS}\right). $$

Hence, Equation (19) shows that the worker group has a non-zero sampling term merely because workers may descend from the same drone; otherwise $var\left({\overline{\delta}}_i\right)$ would average to zero. Finally, diagonal elements for worker groups follow from Equation (18).

### Off-diagonal elements of **D**

Off-diagonal elements of **D** occur only between individuals that derive from the same queen and sire combination and are given by (see above Equation (17)):

20 $$ {\mathbf{D}}_{ij}=\frac{cov\left({\delta}_{FS}\right)}{\sigma_A^2}. $$

### Covariance between sampling terms of full-sibs ***cov***(***δ***_***FS***_)

In honey bees, full-sibs are the offspring of the mating between a queen and a sire. Within a colony, some pairs of workers are full-sibs in the ordinary sense (when they descend from a common queen and a common drone-producing queen, but from different drones) with an additive genetic relationship of ${a}_{XY}=\frac{1}{2}$, ignoring inbreeding. A pair may also descend from the same drone, resulting in ${a}_{XY}=\frac{3}{4}$, or from different drone-producing queens, resulting in ${a}_{XY}=\frac{1+{a}_{SS}}{4}$.

Usually in animal breeding, the covariance between breeding values of relatives is fully accounted for by the pedigree. In general, however, this requires two conditions. The first condition is that, conditional on the pedigree, Mendelian sampling terms of offspring are independent. In honey bees this is not the case for full-sibs, because they may descend from the same drone, in which case their paternal Mendelian sampling terms are identical. The second condition is that the pedigree fully accounts for the contributions of parents to offspring. Usually in animal breeding, this condition is met, because a parent contributes precisely half the genes of an offspring. However in a honey bee pedigree, this condition is not met because the sire is an aggregate of multiple drone-producing queens and the contribution of individual drone-producing queens to offspring varies among the drone-producing queens that constitute a sire. This will occur by chance, even when the *a priori* expected contribution is the same for all drone-producing queens that make-up a sire but the pedigree accounts for only the average contribution of a drone-producing queen to the offspring, which is given by the $\frac{1}{2}{\overline{A}}_s$ in Equations (9) and (14). Variation among drone-producing queens in their contribution to offspring creates a paternal covariance among full-sibs that exceeds the $var\left(\frac{1}{2}{\overline{A}}_s\right)$ that is accounted for by the pedigree, and thus creates a covariance between the δ terms of full-sibs. Thus, the δ terms of full-sibs may be correlated because (i) sibs may descend from the same drone, and (ii) the contribution to offspring may vary among drone-producing queens.

Let *p*_*1*_ denote the probability that two full-sibs descend from the same drone, and *p*_*2*_ the probability that they descend from the same drone-producing queen (including the case where they descend from the same drone, so *p*_*2*_ > *p*_*1*_).

21 $$ \begin{array}{l}\mathrm{Then}, cov\left({\delta}_{FS}\right)={p}_1\frac{1-{F}_s}{4}{\sigma}_A^2+\\ {}\left({p}_2-\frac{1}{S}\right)\frac{\left(1+{F}_s-{a}_{ss}\right)}{4}{\sigma}_A^2.\end{array} $$

The first term in equation (21) arises from the probability that two full-sibs descend from the same drone. The second term arises from variation in the contribution of individual drone-producing queens to offspring, around the expected value of $\frac{1}{S}$. Thus, in the second term, the $-\frac{1}{S}$ term represents subtraction of the covariance already accounted for by the pedigree.

Both *p*_1_ and *p*_2_ depend on variation in contributions of parents to offspring. For *p*_1_, suppose that the *i*^th^ drone contributes a fraction *c*_*D*,*i*_ to the offspring of the queen, so that ${\displaystyle \sum_1^D{c}_{D,i}=1}$, where *D* denotes the number of drones that mate to a queen. Then, the probability that two full-sibs descend from the same drone is ${p}_1={\displaystyle \sum_1^D{c}_{D,i}^2}$. Since ${\overline{c}}_D=\frac{1}{D}$, this can be written as:

22 $$ {p}_1=D{\sigma}_{c_D}^2+\frac{1}{D}, $$

where ${\sigma}_{c_D}^2$ is the variation among the drones that mated to the queen in their contributions to its offspring. This result shows that variation in contributions among drones increases the covariance among full-sibs. Analogously, for drone-producing queens, it follows that:

23 $$ {p}_2=S{\sigma}_{c_S}^2+\frac{1}{S}, $$

where ${\sigma}_{c_S}^2$ is the variation among the drone-producing queens in their contributions to the offspring of the queen (thus ${\displaystyle \sum_{i=1}^S{c}_{S,i}}=1$ and ${\overline{c}}_S=\frac{1}{S}$).

Equations (21) to (23) are valid irrespective of the distribution of the contributions of drones and drone-producing queens to offspring. In other words, *p*_1_ and *p*_2_ do not depend on the details of that distribution, but only on the variance. In practical applications, empirical values for ${\sigma}_{c_D}^2$ and ${\sigma}_{c_S}^2$ may be used. However, when such values are not available, the expected values of ${\sigma}_{c_D}^2$ and ${\sigma}_{c_S}^2$ may be derived under the assumption that the number of offspring of a parent follows a Poisson distribution, which is the default distribution for family size in population biology. Assuming that the number of offspring of a drone follows a Poisson distribution, it follows that (see Appendix 2):

22a $$ {p}_{1, Poisson}=\frac{1}{T}+\frac{1}{D}\approx \frac{1}{D}, $$

where *T* denotes the total number of offspring of a queen. Since *T* is very large, *p*_1_ will be close to $\frac{1}{D}$ when family size follows a Poisson distribution. Moreover, when the number of drones of a single drone-producing queen that mates to the queen follows a Poisson distribution and the number of offspring per drone is large, then it follows that (Appendix 2):

23a $$ {p}_{2, Poisson}\approx \frac{1}{D}+\frac{1}{S}. $$

Substituting those values into Equation (21) yields:

21a $$ cov\left({\delta}_{FS,\  Poisson}\right)=\frac{2-{a}_{ss}}{4D}{\sigma}_A^2. $$

Finally, off-diagonals of ***D*** are obtained from substituting Equation (21a) into Equation (20). Thus, when the number of offspring per parent follows a Poisson distribution, the covariance between Mendelian sampling terms of full-sibs depends only on the relatedness between drone-producing queens (*a*_*ss*_) and on the number of drones mated to a queen. Under the assumption that the number of drones of a single drone-producing queen that mates to the queen follows a Poisson distribution, there is no covariance between sampling deviations of paternal half sibs (*i.e.*, between two offspring of the same sire but of a different queen; see Discussion). Whether the assumption of a Poisson distribution is realistic will be addressed in the Discussion.

In method BER, ${p}_1=\frac{1}{D}$ was used, assuming equal contribution of each drone to the progeny. To obtain *p*_*2*_, a Poisson distribution was not assumed but the total probability that full-sibs descend from different drones i.e., $1-{p}_1=1-\frac{1}{D}$, was partitioned into a fraction $\frac{1}{S}$ for the same drone-producing queen, and a fraction $1-\frac{1}{S}$ for different drone-producing queens. In that case, the probability that two full-sibs descend from the same drone-producing queen equals $\frac{1}{D}$ (the probability that two progeny descend from the same drone) plus $\left(1-\frac{1}{D}\right)\frac{1}{S}$ (the probability that two progeny descend from the same drone-producing queen but from two different drones), which gives a total probability of:

23b $$ {p}_{2, BER}=\frac{S+D-1}{DS}, $$

which differs from the *p*_2_ for a Poisson distribution by an amount equal to $\frac{-1}{DS}$. Replacing *p*_2_ in Equation (21) by *p*_2,*BER*_ yields:

21b $$ \begin{array}{l} cov\left({\delta}_{FS,\  BER}\right)=\\ {}\frac{1-{F}_s}{4D}{\sigma}_A^2+\frac{\left(1+{F}_s-{a}_{ss}\right)\left(S-1\right)}{4 DS}{\sigma}_A^2\end{array} $$

Note that the BER method does not implement off-diagonal elements in **D** (see below); here, we merely present Equation (21b) to show the outcome of *cov*(*δ*_*FS*_) for the *p*_2_ proposed by [5]. Note that for S-1 approaching S, Equation (21b) approaches (21a).

### Construction of **D** and **D**^− 1^

Calculation of the elements of **D** requires additive genetic relationships between drone-producing queens, *a*_*ss*_, and inbreeding coefficients, *F*. These values can be obtained recursively when proceeding in the pedigree, starting with the oldest individuals. For sires from the base generation of the pedigree, it is reasonable to take *a*_*ss*_ = 0, because their dams can be considered as unrelated just like the drones they are mated to. For later generations, *a*_*ss*_ builds up stepwise according to:

24 $$ \begin{array}{l}{a_{ss}}_i=\frac{1+{F}_d}{4}+\frac{1}{2}{p}_1+\frac{1}{4}\left({p}_2-{p}_1\right)\left(1+{F}_s\right)+\\ {}\frac{1}{4}\left(1-{p}_2\right){a}_{s{s}_{i-1}} + \frac{a_{sd\ }}{2}\ .\end{array} $$

In this equation, the first term represents the additive genetic relationship between drone-producing queens because they descend from the same dam, the second term relates to the case when they descend from the same drone, which has probability *p*_1_ and a paternal relatedness of $\frac{1}{2}$, the third term relates to the case when they descend from the same drone-producing queen but from a different drone, which has probability (*p*_2_ − *p*_1_) and a paternal relatedness of $\frac{1}{4}\left(1+{F}_S\right)$, the fourth term relates to the case when they descend from different drone-producing queens, which has probability (1 − *p*_2_) and a paternal relatedness of $\frac{a_{ss}}{4}$, and the last term accounts for the additive genetic relationship between dam and sire of the drone-producing queens.

With a Poisson distribution for the numbers of drones and drone-producing queens mating to the queen, ${p}_1\approx \frac{1}{D}$ and ${p}_2\approx \frac{1}{D}+\frac{1}{S}$ (Equations (22a) and (23a)), so that Equation (24) becomes:

24a $$ \begin{array}{l}{a}_{s{s}_i,\kern0.5em  Poisson}=\frac{1+{F}_d}{4}+\frac{1}{2D}+\frac{1+{F}_s}{4S}+\\ {}\frac{\left( DS-D-S\right){a}_{s{s}_{i-1}}}{4 DS}+\frac{a_{sd\ }}{2}.\end{array} $$

When substituting the *p*_2_ of BER, its expression being given by Equation (23b) here, into Equation (24) we get:

24b $$ \begin{array}{l}{a}_{s{s}_i,\  BER}=\frac{1+{F}_d}{4}+\frac{1}{2D}+\frac{\left(D-1\right)\left(1+{F}_s\right)}{4 DS}+\\ {}\frac{\left(D-1\right)\left(S-1\right){a_{ss}}_{i-1}}{4 DS}+\frac{a_{sd}}{2}\end{array} $$

Inbreeding coefficients can be derived from the additive genetic relationship between the sire and the dam of individual *i* as:

25 $$ {F}_i=\frac{1}{2}{a}_{sd}. $$

As a result, **D** is a block-diagonal matrix, each block representing the offspring of a single queen, *i.e.* the combination of a queen and a sire. Chronologically, such a block starts with a single individual, being the worker group that descends from that queen. When the queen is selected to breed new queens, the queens in its progeny will be added to the block. Moreover, when the queen is selected to breed drone-producing queens, then one or more sires will be added to the block. The size of a block, therefore, equals 1 plus the number of queens plus the number of sires that descend from the mother queen. Thus, a block contains at maximum three distinct diagonal values, one for the worker group, one for queens, and one for sires. All off-diagonals within a block are equal, and equal to the diagonal element for the worker group (Equation (20)). Off-diagonals outside blocks are 0.

Since **D** is a block-diagonal matrix, the inverse of **D** is also a block-diagonal matrix, each block being the inverse of the corresponding block of **D**. Since blocks of **D** have a specific structure, with at maximum three distinct values, **D**^− 1^ can be obtained analytically, *e.g.*, with the help of equation-solving software such as Mathematica [16]. However, since blocks of **D** can have different numbers of queens and sires, there are multiple analytical solutions, each of which is a complicated expression. Therefore, since the size of the blocks is usually small, numerical inversion of each block is easy and more practical and, thus, we do not present the analytical inversion of **D** here.

### The Bienefeld, Ehrhardt and Reinhardt (BER) method [5]

The main methodological problem addressed in [5,10] is that the additive genetic relationship that can be attributed to the sire differs between two workers in *the same* colony versus two workers in *different* colonies. This difference arises because workers within a colony partly descend from the same drone, whereas workers in different colonies must derive from different drones. In the BER method, these two additive genetic relationships are replaced by a single additive genetic relationship, the square root of which is the path coefficient *q* between a sire and the workers descending from this sire. Consequently, breeding values are estimated using:

26 $$ {A}_i=\frac{1}{2}{A}_d+q{A}_s+{\delta}_i. $$

The approach used by Bienefeld, Ehrhardt and Reinhardt [5] consists of two steps. In the first step, the asymptotic value of the additive genetic relationship between full-sibs is calculated by ignoring inbreeding and the additive genetic relationship between dam and sire. The asymptotic value is obtained by solving the equilibrium condition ${a}_{s{s}_i}={a}_{s{s}_{i-1}}$ in Equation (24b), together with *a*_*sd*_ = *F*_*s*_ = *F*_*d*_ = 0. Numerically, the asymptotic value of *a*_*sd*_, denoted by *a*_*FS*_, is approached closely within a few generations [5]. Then, the paternal component of the additive genetic relatedness between workers in the same colony is obtained by subtracting the maternal component, ${a}_{pFS}={a}_{FS}-\frac{1}{4}$. Because full-sibs can descend from the same drone, the resulting value differs from the additive genetic relationship between paternal half sibs (i.e., workers in different colonies), which is ${a}_{pHS}=\frac{1+\left(S-1\right){a}_{FS}}{4S}$. In the second step of the BER method, both relationships are replaced by their mean, i.e.

27 $$ a=\frac{a_{FS}-\frac{1}{4}+{a}_{pHS}}{2}, $$

and the additive genetic relationship between a worker and its sire is calculated as the square root of this mean, i.e.

28 $$ q=\sqrt{\frac{a_{FS}-\frac{1}{4}+{a}_{pHS}}{2}}\ . $$

Then, **M** and **D** follow from the model in Equation (26). In **M**, the row for an offspring has $\frac{1}{2}$ in the column for its mother (the queen) and *q* in the column for its father (the sire). Matrix **D** is assumed to be diagonal with elements ${\sigma}_{\delta_i}^2/{\sigma}_A^2$ that equal:

29 $$ \begin{array}{l}{\mathbf{D}}_{ii}=1+\left(\frac{1}{2}-q\right){a}_{ds}-\frac{1}{4}\left(1+{F}_d\right)-\\ {}{q}^2\left(1+{F}_s\right),\end{array} $$

when both parents are known (Appendix 1). This result shows that **D**_*ii*_ includes not only the Mendelian sampling variance but also a component due to the difference between *q* and $\frac{1}{2}$.

In the BER method, both the sire and the worker group are treated as single individuals, not as virtual individuals that consist of a group of individuals. As a consequence, the diagonal elements of **D** are neither affected by the number of drone-producing queens nor by the number of drones involved in mating, so that the sampling variances are computed in the same manner as those of queens.

Note that in [13], the breeding value of an individual is described as:

30 $$ {A}_i=\frac{1}{2}{A}_d+q{A}_s+\left(\frac{1}{2}-q\right){\overline{A}}_s+{\delta}_i, $$

where the term $\left(\frac{1}{2}-q\right){\overline{A}}_s$ is a correction to account for the fact that offspring inherit less than 50% of their genes from the sire in Equation (26). In the current implementation of breeding value estimation in the Beebreed program, ${\overline{A}}_s$ represents the average breeding value of sires of a particular year (Ehrhardt K, 2013, personal communication). This correction is essential to properly account for genetic trend when parents are selected across years.

### Simulation

The purpose of the simulation was to study properties of estimated breeding values from the BER and BB methods, and to compare the estimated breeding values. In the simulation, breeding values are generated for three types of individuals, queens, sires and worker groups, and subsequently the phenotypes for colonies are generated.

Table 1 illustrates the selection scheme. Two-year-old tested queens produce the next generation of virgin queens. Just after birth, these virgin queens are mated to sires. The sires descend from queens that are three years old at birth of the virgin queens. In the actual Beebreed program, the dams may also produce offspring at an older age but Table 1 illustrates the most frequent situation. Table 1 also illustrates that sires on mating stations (or with artificial insemination) are mated to groups of full-sib queens. At two years of age each queen produces a colony observation that is added to the data to estimate breeding values.

**Table 1:** **Simplified selection cycle in the honey bee selection programme**

**Year**	**Queens**	**Sires**
t		Selection of queens to produce drone-producing queens (sires); birth of sires
t + 1	Selection of queens to produce full-sib groups of queens to be mated to sires	Use of sires for mating to groups of queens, each group being the progeny of a selected queen
t + 2	Test of colonies of the set of full-sibs born in t + 1	
t + 3	Selection of queens and birth of next-generation queens to be mated to sires born in t + 2	Selection of queens to produce drone-producing queens

The basic simulation starts with NSY (number of sires per year) base sires generated in years 1, 2 and 3, and NQY (number of queens per year) base queens generated in years 2 and 3. The base queens were simulated as full-sib groups with NQ (number of full-sibs per group) individuals each because that is also the structure in future generations. Sires from years 1 and 2 are mated randomly to queens in years 2 and 3 with an equal number of NFQ mates for each sire, each producing NQ full-sibs. From year 4, the NSY queens with the highest estimated breeding value according to Equation (2) are selected to produce the next generation of sires. The NSYxNFQ queens with the highest estimated breeding value are selected to produce NQ queens each. Allocation of mates (sires) to queens is random, but each queen within a full-sib group is mated to the same sire and each sire is mated to the same number of queens. The queens that will produce sires are therefore selected NSY out of NQY, and the queens that will produce queens are selected 1 out of NQ.

Two breeding values are simulated for each individual, a breeding value for the worker effect and a breeding value for the queen effect. To allow for a correlation between these two breeding values, random samples for an individual are taken from a bivariate normal distribution (using the function mvrnorm from the package MASS in R; http://cran.r-project.org/package=MASS). Because Mendelian sampling terms are correlated between offspring of the same queen, sampling terms were constructed as the sum of two independent components: a component specific to each individual and a component common to all offspring of the same queen.

For the *i*^th^ queen belonging to the *d*^th^ dam family, the two breeding values were generated as:

31 $$ \begin{array}{l}\left[\begin{array}{c}\hfill {A}_i^W\hfill \\ {}\hfill {A}_i^Q\hfill \end{array}\right]=\\ {}\frac{1}{2}\left[\begin{array}{c}\hfill {A}_d^W+{\overline{A}}_s^W\hfill \\ {}\hfill {A}_d^Q+{\overline{A}}_s^Q\hfill \end{array}\right]+\left[\begin{array}{c}\hfill {n}_d^W\ \sqrt{cov\left({\delta}_{FS,d}^W\right)}\hfill \\ {}\hfill {n}_d^Q\ \sqrt{cov\left({\delta}_{FS,d}^Q\right)}\hfill \end{array}\right]+\\ {}\left[\begin{array}{c}\hfill {n}_i^W\ \sqrt{var\left({\delta}_i^W\right)- cov\left({\delta}_{FS,d}^W\right)}\ \hfill \\ {}\hfill {n}_i^Q\ \sqrt{var\left({\delta}_i^Q\right)- cov\left({\delta}_{FS,d}^Q\right)}\hfill \end{array}\right], \end{array} $$

where $\left({n}_d^W,{n}_d^Q\right)$ is a sample from a standard bivariate normal distribution with correlation *r*_*G*_, which was sampled once for each dam family (hence the subscript *d*). The $\left({n}_i^W,{n}_i^Q\right)$ is another sample from the same distribution, independent of the previous, which was sampled once for each individual queen (hence the subscript *i*). Thus, the second term on the right-hand side of Equation (31) is common to all offspring of the same dam family, whereas the third term is specific to each individual offspring. The *cov*(*δ*_*FS*,*d*_) denotes the sampling variance common to all offspring of dam family *d*, superscripts *Q* and *W* denoting the queen and worker effect respectively, and was obtained from Equation (21b), assuming proportional contributions of sires and drones according to the BER method. The term *var*(*δ*_*i*_) − *cov*(*δ*_*FS*,*d*_) in Equation (31) represents the remaining sampling variation for an individual queen after subtracting the variance common to all offspring of the same dam, i.e. *cov*(*δ*_*FS*,*d*_). The *var*(*δ*_*i*_) in Equation (31) was taken from Equation (13).

For the *i*^th^ sire belonging to the *d*^th^ dam family, the two breeding values were generated as

32 $$ \begin{array}{l}\left[\begin{array}{c}\hfill {\overline{A}}_i^W\hfill \\ {}\hfill {\overline{A}}_i^Q\hfill \end{array}\right]=\\ {}\frac{1}{2}\left[\begin{array}{c}\hfill {A}_d^W+{\overline{A}}_s^W\hfill \\ {}\hfill {A}_d^Q+{\overline{A}}_s^Q\hfill \end{array}\right]+\left[\begin{array}{c}\hfill {n}_d^W\ \sqrt{cov\left({\delta}_{FS,d}^W\right)}\hfill \\ {}\hfill {n}_d^Q\ \sqrt{cov\left({\delta}_{FS,d}^Q\right)}\hfill \end{array}\right]+\\ {}\left[\begin{array}{c}\hfill {n}_i^W\ \sqrt{var\left({\overline{\delta}}_i^W\right)- cov\left({\delta}_{FS,d}^W\right)}\ \hfill \\ {}\hfill {n}_i^Q\ \sqrt{var\left({\overline{\delta}}_i^Q\right)- cov\left({\delta}_{FS,d}^Q\right)}\hfill \end{array}\right], \end{array} $$

where only the last term differs from Equation (31) and $var\left({\overline{\delta}}_i\right)$ was taken from Equation (17).

For worker groups, the individual sampling deviation is practically 0 because of the large numbers of individuals in a worker group (Equation (19)). Since a queen has only a single worker group, in Equation (32) subscript *i* can be replaced by *w*, so that the two breeding values for the worker group belonging to the *d*^th^ dam family were generated as:

33 $$ \left[\begin{array}{c}\hfill {\overline{A}}_w^W\hfill \\ {}\hfill {\overline{A}}_w^Q\hfill \end{array}\right]=\frac{1}{2}\left[\begin{array}{c}\hfill {A}_d^W+{\overline{A}}_s^W\hfill \\ {}\hfill {A}_d^Q+{\overline{A}}_s^Q\hfill \end{array}\right]+\left[\begin{array}{c}\hfill {n}_d^W\ \sqrt{cov\left({\delta}_{FS,d}^W\right)}\hfill \\ {}\hfill {n}_d^Q\ \sqrt{cov\left({\delta}_{FS,d}^Q\right)}\hfill \end{array}\right] $$

When a sire, queen or worker descended from the same dam, then the values of $\left({n}_d^W,{n}_d^Q\right)$ in Equations (31) through (33) are identical for those individuals.

Based on Equation (1), colony observations were generated as:

34 $$ {P}_c={\overline{A}}_w^W+{A}_d^Q+{n}_c{\sigma}_E, $$

where *n*_*c*_ is a sample from a univariate standard normal distribution.

Using the simulated data and pedigree, the inverse numerator relationship matrix **A**^− 1^ (Equation (8)) was created using either method BB or method BER, and breeding values were estimated by solving Equation (4). The criterion to select queens to produce the next generation of queens and sires for method BB is given by Equation (2). For method BER, the factor of $\frac{1}{2}$ for the estimated breeding value of the sire in Equation (2) is replaced by *q*.

To evaluate the effect of selection using the two methods, we analysed the effect on the true breeding value of unfertilized queens (the breeding goal), which were simulated as:

35 $$ \begin{array}{l}{A}_Q^W+{A}_Q^Q=\frac{1}{2}\left({A}_d^W+{A}_d^Q\right)+\\ {}\frac{1}{2}\left({\overline{A}}_s^W+{\overline{A}}_s^Q\right)+{\delta}_Q,\end{array} $$

which is the sum of the dam’s and the sire’s breeding value for worker and queen effects, plus Mendelian sampling. The Mendelian sampling consists of a term common to all offspring of the pair of dam and sire, using the common values for ${n}_d^W$ and ${n}_d^Q$, and a residual sampling term, as in Equation (31).

Parameter values used in the simulation were ${\sigma}_{A^W}^2$ = 1, ${\sigma}_{A^Q}^2$ = 0.5, ${\sigma}_E^2$ = 2 and *r*_*G*_ = −0.5, which are in line with estimates reported by [13]. Based on [5], the number of drone-producing queens that constitute a sire (S) was equal to 8 and the number of drones mating to a queen (D) was equal to 12. We analysed the simulated data for a small example, in which the only fixed effect was the mean. NSY was equal to 5, the number of full-sib groups to which a sire is mated (NFQ) was equal to 5 and NQ was fixed at 3. We simulated 20 years of data, including colony performance of queens born in year 20.

First, the properties of estimated breeding values (EBV) were investigated using 1000 replicated schemes without selection. The quality of EBV was judged by the regression coefficient of the true (*i.e.,* simulated) breeding value (TBV) on the EBV and by the correlation coefficient between TBV and EBV in year 20. We chose those criteria because the regression coefficient of TBV on EBV should be equal to 1 with BLUP (best linear unbiased prediction), while response to selection on EBV is proportional to the correlation coefficient. We did not implement the correction factor $\left(\frac{1}{2}-q\right){\overline{A}}_s$ from Equation (30), since this does not affect results because regression and correlation coefficients were calculated within one generation. Second, we compared response to selection between the two methods, again using 1000 replicates. Because selection took place within years (non-overlapping generations), we did not implement the correction factor of Equation (30) here either, since it did not affect results.

## Results

Table 2 gives the regression coefficients of TBV (simulated) on EBV from 1000 replicates of simulation. The results with method BB were according to theory: regression coefficients of TBV on EBV were very close to 1, not only for year 20, but also for preceding years (results not shown). With method BER, regression coefficients deviated from 1 and, in early years, from the stable values reached in later years. For queens, the regression coefficient for the EBV for the queen effect was larger than 1, which means that the variance in EBV was too small, i.e. positive TBV were underestimated and negative TBV were overestimated. Thus, the BER method shrinks the EBV too much towards the mean. Also the regression coefficients for sires in year 20 were larger than 1, although with a large standard deviation. The regression coefficients for colonies were much lower than 1, which is primarily a variance issue: in the BER method, colonies are treated as single individuals so that their variance is taken to be equal to that of a queen, while in fact it is much smaller due to the averaging of Mendelian sampling terms.

**Table 2:** **Regression coefficients of true breeding values on estimated breeding values** ^**1**^ **obtained from two methods (BB and BER)**

	**BB**		**BER**	
	**Worker effect**	**Queen effect**	**Worker effect**	**Queen effect**
Queens	0.971 (0.022)	0.998 (0.014)	1.061 (0.025)	1.160 (0.016)
Sires	1.088 (0.080)	1.025 (0.056)	1.148 (0.076)	1.175 (0.071)
Colonies	0.998 (0.017)	1.000 (0.019)	0.423 (0.007)	0.699 (0.024)

^1^Values refer to year 20; standard errors are given in brackets; BB is the method developed in this paper and BER is the method developed by Bienefeld, Ehrhardt and Reinhard [5].

Response to selection depends on the accuracy of the estimated values for the breeding objective given by Equation (2), in pairs of queens and sires that are candidates to be selected to breed future queens. To get an impression of possible responses to selection using the two methods, we studied the correlations between the TBV (simulated) and EBV for the breeding objective. Results are in Table 3 and show that, for this simple example, the correlations with methods BB and BER were fairly similar. Across years, correlations differed by nearly 10% between both methods. Although the EBV obtained with the BER method were not unbiased, while those with the BB method were, the animals ranked similarly.

**Table 3:** **Correlations between the true and estimated breeding values and cumulative responses to selection with two breeding value estimation methods (BB and BER)** ^**1**^

	**Correlation** ^**2**^		**Cumulative response to selection** ^**3**^	
**Year**	**BB**	**BER**	**BB**	**BER**
2	0.1845	0.1845	0.0012	−0.0025
3	0.1884	0.1884	0.0006	−0.0023
4	0.2244	0.2170	0.0886	0.0842
5	0.2744	0.2581	0.2463	0.2404
6	0.2771	0.2613	0.3114	0.2934
7	0.2980	0.2741	0.4732	0.4534
8	0.3014	0.2756	0.6159	0.5819
9	0.3015	0.2737	0.7106	0.6859
10	0.3161	0.2855	0.8679	0.8296
11	0.3037	0.2768	0.9823	0.9461
12	0.3072	0.2765	1.1052	1.0664
13	0.3120	0.2849	1.2296	1.1921
14	0.3098	0.2778	1.3546	1.3064
15	0.3118	0.2828	1.4771	1.4306
16	0.3048	0.2795	1.6092	1.5377
17	0.3061	0.2794	1.7243	1.6577
18	0.3075	0.2785	1.8564	1.7770
19	0.3104	0.2821	1.9632	1.8897
20	0.3084	0.2782	2.0894	2.0086

^1^Correlations were calculated from schemes in which no selection was practiced; ^2^standard errors are about 0.004; ^3^standard errors increase from about 0.0040 in year 2 to about 0.0110 in year 20; BB is the method developed in this paper and BER is the method developed by Bienefeld, Ehrhardt and Reinhard [5].

Responses to selection are also in Table 3. The annual responses to selection started slowly in early years and remained somewhat irregular in later years. There was a strong similarity in results for the two methods in that respect. This irregularity is caused by the structure of the simulation. The simulation started with the simulation of base sires in years 1 to 3 and base queens in years 2 and 3. A first batch of offspring (born in year 4) is produced from base sires of year 1 and base queens of year 2 and a second batch (born in year 5) from base sires of year 2 and base queens of year 3. Genetically, progeny of these batches of offspring mixed in later years due to the fact that the dams of the sires were three years old at birth of the next generation of queens, while the dams were two years old at birth of the next generation of queens. However, this mixing developed fairly slowly and was delayed by the fact that pairs of queens and sires were selected to produce the next generation. Similar results have been observed in simulations of breeding schemes with overlapping generations in dairy cattle [17]. The cumulative selection response differed little between the two methods BB and BER but were 5% higher with method BB compared to method BER in years 8 to 10, and 4% higher in years 18 to 20.

## Discussion

In this paper, we derived a method to calculate the relationship matrix and its inverse for honey bee populations, which is required to estimate breeding values and genetic parameters. The situation in honey bees differs from the usual situation in farm animal breeding, because of the honey bees’ mode of reproduction. The first major difference is that two full-sibs may carry identical paternal gametes. This occurs because sires (drone-producing queens) produce drones which may be considered as flying gametes that produce many identical sperm cells. Because a drone can mate to a single queen only, paternal half-sibs always carry different paternal gametes. Consequently, the paternal contribution to the additive genetic relationship between full-sibs differs from that between half-sibs, which results in a block diagonal **D** matrix of covariances between Mendelian sampling terms. Off-diagonals of those blocks equal the covariance between sampling terms of full-sibs. The second difference is that selection candidates (queens) are mated early in life, before they can be selected as parents. As a consequence, selection is not of individual dams but of matings from which breeding stock can be produced after the estimation of breeding values. Thus, the selection target is the breeding value of a future queen from this mating, which equals half the breeding values of both mates plus the part of Mendelian sampling that is common to all progeny of these mates. This also implies that the EBV of such a future queen equals the EBV of the colony. Another difference from traditional animal breeding is that the “father” of a queen is usually unknown because the drones that mate with the queen come from multiple drone-producing queens. In this context, our work follows that of Dempfle [8], who discussed the consequences of mixed semen for the estimation of breeding values, rather than focussing on the haploid nature of the drones [6,7].

Equation (21) gives a general expression for the covariance of the Mendelian sampling terms of full-sibs. This covariance depends on the variance of the number of offspring per drone (Equation (22)) and the variance of the number of drones per drone-producing queen (Equation (23)). Without further knowledge, a Poisson distribution of family sizes is a common choice, which leads to Equation (21a). Numerically, this equation differs very little from Equation (21b), which results from substituting the probabilities from the BER method (Equation (23b)) into Equation (21), but these probabilities do not have a theoretical basis. However in reality, the assumption of a Poisson distribution of family sizes does not seem to hold, since a review of the literature [18] suggests that the proportion of progeny descending from different drones deviates from Poisson. Furthermore, results of an experiment using drone-producing colonies each producing a similar number of drones, suggested that drone-producing queens that contribute a higher proportion of drones to matings also produce drones with a higher proportion of offspring in colonies [19]. The Poisson distribution arises when variation in contributions is entirely by chance, *i.e.*, when a priori the expected number of offspring is equal for each drone-producing queen and for each drone. When there are systematic differences between drone-producing queens or drones in the expected number of offspring, then the variance in contributions will be larger than in the case of a Poisson distribution, which implies a larger covariance between sampling terms of full-sibs. Numerically, this effect may be neutralised by assuming a smaller number of drones, *i.e.*, by using an effective number of drones rather than the actual number of drones. Note that in this context, [5] used a number of drones equal to 12, while a consensus number is around 16 [18]. When assuming a Poisson distribution, the covariance between Mendelian sampling terms for half sibs is 0. This is, however, not true if some drone-producing queens are systematically more successful to contribute drones to matings [19].

In practice, in the Beebreed program, inbreeding coefficients are computed for possible planned matings that are not yet included in the pedigree (http:www.beebreed.eu). Efficient methods to compute inbreeding coefficients have been derived [20,21], based on [15]. These methods exploit the fact that the **D** matrix is a diagonal matrix. We derived a modification to [15], which takes into account the fact that the **D** matrix contains off-diagonal elements in the bee breeding case. This method, however, requires the whole pedigree of an individual to be searched for the occurrence of parents that are full-sibs, which may be very time-consuming. As an alternative, the **A** matrix of the pedigree may be kept in memory such that the required inbreeding coefficients can be used in Equation (25).

In the development of the methods and analyses presented here, we used the current mating system applied in the Beebreed program as a starting point. This implies that drone-producing queens are full-sibs from a shared dam and sire. That may not be the case for parts of the pedigree or for other programs. For those cases, we suggest to include the individual drone-producing queens in the pedigree, with diagonal elements in **D** equal to those of individual queens, combining Equations (13) and (10). Elements in **M** then need to be adapted to reflect the fractions that are contributed by these individual drone-producing queens. Without prior knowledge on these fractions, we suggest to use equal fractions as an approximation, although this may not be true in reality [18,22].

## Conclusions

We have presented methodology to construct the relationship matrix and its inverse for honey bee populations, which is required in the mixed model equations used for the estimation of breeding values and genetic parameters. The method allows for different assumptions on the contribution of drones and drone-producing queens to offspring, and is exact if those assumptions are correct. The method yields EBV that are unbiased predictors of TBV. We also carried out an exploratory comparison with the BER method [5] that is currently used in practice and weighs information on relatives, differently. Although EBV obtained with the BER method were biased, selection candidates were ranked similar to those of our method and the response to selection was only slightly lower than with our method. This suggests that suboptimal weighting of information from relatives has limited impact on the ranking of selection candidates. It remains to be seen whether this conclusion extends to the estimation of genetic parameters.

## Appendix 1

### Variance of Mendelian sampling terms

In this Appendix, first we derive Equation (13), the Mendelian sampling variance for queens with known parents for method BB. Subsequently, we derive the variances when parents are unknown, for queens and sires. Finally, we repeat all steps for method BER. Equation numbers between brackets refer to equations in the main text.

### BB method

The model describing the breeding value of a queen is:

9 $$ {A}_i=\frac{1}{2}{A}_d+\frac{1}{2}{\overline{A}}_s+{\delta}_i, $$

such that the variance of the breeding value can be written as:

11 $$ \begin{array}{l}{\sigma}_A^2\left(1+{F}_i\right)=\frac{1}{4}{\sigma}_A^2\left(1+{F}_d\right)+\frac{1}{4} var\left({\overline{A}}_s\right)+\\ {}\frac{1}{2} cov\left({A}_d,{\overline{A}}_s\right)+ var\left({\delta}_i\right).\end{array} $$

Because $\frac{1}{2} cov\left({A}_d,{\overline{A}}_s\right)={F}_i{\sigma}_A^2$, and furthermore:

$$ \begin{array}{l} var\left({\overline{A}}_s\right)=\\ {}\frac{1}{S^2}\left(S{\sigma}_A^2\left(1+{F}_s\right)+S\left(S-1\right){a}_{ss}{\sigma}_A^2\right)=\\ {}\frac{\sigma_A^2}{S}\left(\left(1+{F}_s\right)+\left(S-1\right){a}_{ss}\right),\end{array} $$

it follows that:

$$ \begin{array}{l} var\left({\delta}_i\right)=\\ {}{\sigma}_A^2-\frac{1}{4}{\sigma}_A^2\left(1+{F}_d\right)-\frac{1}{4} var\left({\overline{A}}_s\right)=\\ {}{\sigma}_A^2-\frac{1}{4}{\sigma}_A^2\left(1+{F}_d\right)-\frac{\sigma_A^2}{4S}\left(1+{F}_s\right)-\\ {}\frac{\sigma_A^2}{4S}\left(S-1\right){a}_{ss}.\end{array} $$

To rearrange this to a biologically useful form we replace:

$$ \begin{array}{l}\frac{\sigma_A^2}{4S}\left(1+{F}_s\right)=\frac{S{\sigma}_A^2}{4S}\left(1+{F}_s\right)-\\ {}\frac{\left(S-1\right){\sigma}_A^2}{4S}\left(1+{F}_s\right),\end{array} $$

which gives:

13 $$ \begin{array}{l} var\left({\delta}_i\right)=\\ {}{\sigma}_A^2\left(1-\frac{1}{4}\left(1+{F}_d\right)-\frac{1}{4}\left(1+{F}_s\right)\right)+\\ {}\frac{1}{4}{\sigma}_A^2\frac{\left(S-1\right)}{S}\left(1+{F}_s-{a}_{ss}\right)=\\ {}{\sigma}_A^2\left(\frac{1}{2}-\frac{1}{4}{F}_d-\frac{1}{4}{F}_s\right)+\\ {}\frac{1}{4}{\sigma}_A^2\frac{\left(S-1\right)}{S}\left(1+{F}_s-{a}_{ss}\right)=\\ {}\frac{1}{4}{\sigma}_A^2\left(1-{F}_d\right)+\frac{1}{4}{\sigma}_A^2\left(1-{F}_s\right)+\\ {}\frac{1}{4}{\sigma}_A^2\frac{\left(S-1\right)}{S}\left(1+{F}_s-{a}_{ss}\right).\end{array} $$

When both parents are unknown the following situations can be distinguished:

1. A queen with unknown parents (base queen). In that case, *A*_*i*_ = *δ*_*i*_ and so $var\left({\delta}_i\right)={\sigma}_A^2$.
2. A sire with unknown parents (base sire). In that case, ${\overline{A}}_i={\overline{\delta}}_i$ and $var\left({\overline{\delta}}_i\right)=\frac{\sigma_A^2}{S}$, because the drone-producing queens constituting the sire are assumed to be unrelated.
3. An individual with a known dam but an unknown sire. In that case, ${A}_i=\frac{1}{2}{A}_d+{\delta}_i$ and ${\sigma}_A^2=\frac{1}{4}\left(1+{F}_d\right){\sigma}_A^2+{\sigma}_{\delta_i}^2$ such that $var\left({\delta}_i\right)=\frac{1}{2}{\sigma}_A^2+\frac{1}{4}\left(1-{F}_d\right){\sigma}_A^2$.
4. An individual with an unknown dam, but a known sire. Then, ${A}_i=\frac{1}{2}{\overline{A}}_s+{\delta}_i$ and ${\sigma}_A^2=\frac{1}{4} var\left({\overline{A}}_s\right)+ var\left({\delta}_i\right)=\frac{\sigma_A^2}{4S}\left(\left(1+{F}_s\right)+\left(S-1\right){a}_{ss}\right) + var\left({\delta}_i\right)$ and after some rearrangement: $var\left({\delta}_i\right)=\frac{1}{2}{\sigma}_A^2+\frac{1}{4}{\sigma}_A^2\left(1-{F}_s\right)+\frac{1}{4}\frac{\left(S-1\right){\sigma}_A^2}{S}\left(1+{F}_s-{a}_{ss}\right)$.
5. A sire with an unknown dam and a known sire. This is very unlikely because it implies that a sire is bred from a dam for which neither pedigree nor breeding values are available. Nevertheless, in that case ${\overline{A}}_i=\frac{1}{2}{\overline{A}}_s+{\delta}_i$ and ${\overline{\delta}}_i=\frac{1}{S}{\displaystyle \sum {\delta}_i}$, as when both parents are known with *δ*_*i*_ as under point 4 of this paragraph, such that $var\left({\overline{\delta}}_i\right)=\frac{var\left({\delta}_i\right)}{S}+\frac{S-1}{S} cov\left({\delta}_i,{\delta}_j\right)$ where *var*(*δ*_*i*_) is as under point 4 and $cov\left({\delta}_i,{\delta}_i\right)=\frac{1}{4D}\left(1-{F}_S\right){\sigma}_A^2$ as before, when both parents are known. Therefore, $var\left({\overline{\delta}}_i\right)=\frac{\sigma_{\delta_i}^2}{S}+\frac{S-1}{4 SD}\left(1-{F}_S\right){\sigma}_A^2$.
6. A sire with a known dam and an unknown sire. This also seems odd. Nevertheless, in that case ${\overline{A}}_i=\frac{1}{2}{A}_d+{\delta}_i$, and ${\overline{\delta}}_i=\frac{1}{S}{\displaystyle \sum {\delta}_i}$ with *δ*_*i*_ as under point 3. Now, $var\left({\overline{\delta}}_i\right)=\frac{var\left({\delta}_i\right)}{S}$, with *var*(*δ*_*i*_) as under point 3. Covariances between *δ*_*i*_ and *δ*_*j*_ are equal to 0 because no covariance due to drones is involved.

### BER method

The model that describes the breeding value of a queen, and also of a sire or colony equals:

19 $$ {A}_i=\frac{1}{2}{A}_d+q{A}_s+{\delta}_i, $$

Therefore

2a $$ \begin{array}{l}{\sigma}_A^2\left(1+{F}_i\right)=\frac{1}{4}{\sigma}_A^2\left(1+{F}_d\right)+\\ {}{q}^2{\sigma}_A^2\left(1+{F}_s\right) + qcov\left({A}_d,{A}_s\right)+ var\left({\delta}_i\right).\end{array} $$

Note that $cov\left({A}_d,{A}_s\right)={a}_{ds}{\sigma}_A^2=2{\sigma}_A^2{F}_i$. Taking this into account it follows that:

$$ \begin{array}{l}\left(1+\frac{1}{2}{a}_{ds}\right){\sigma}_A^2=\frac{1}{4}{\sigma}_A^2\left(1+{F}_d\right)+\\ {}{q}^2{\sigma}_A^2\left(1+{F}_s\right) + q{a}_{ds}{\sigma}_A^2+ var\left({\delta}_i\right)\end{array} $$

and

$$ \begin{array}{l}\  var\left({\delta}_i\right)=\left(1+\left(\frac{1}{2}-q\right){a}_{ds}\right){\sigma}_A^2-\\ {}\left(\frac{1}{4}\left(1+{F}_d\right)-{q}^2\left(1+{F}_s\right)\right){\sigma}_A^2.\end{array} $$

When neither the dam nor the sire is known $var\left({\delta}_i\right)={\sigma}_A^2$.

In the case when only the dam is known, ${A}_i=\frac{1}{2}{A}_d+{\delta}_i$ and $var\left({\delta}_i\right)=\left(1-\frac{1}{4}\left(1+{F}_d\right)\right){\sigma}_A^2$. In the case when only the sire is known, *A*_*i*_ = *qA*_*s*_ + *δ*_*i*_, and $var\left({\delta}_i\right)=\left(1-{q}^2\left(1+{F}_s\right)\right){\sigma}_A^2$.

## Appendix 2

Here, we derive the probability *p*_1_ that two offspring of a queen descend from the same drone, and the probability *p*_2_ that two offspring descend from the same drone-producing queen, under the assumption that the number of drones per drone-producing queen and the number of offspring per drone follow a Poisson distribution. Take the average number of drones per drone-producing queen to be equal to *D*_*S*_ and the average number of offspring per drone to be equal to *N*_*D*_. Furthermore, take the total number of offspring per sire to be equal to *T*, such that *T* = *SD*_*S*_*N*_*D*_ and the number of offspring per drone-producing queen to be equal to *N*_*S*_.

The average number of drones is equal to *D*, such that the average number of offspring per drone equals $\frac{T}{D}={N}_D$. Furthermore, because the number of offspring per drone follows a Poisson distribution:

$$ var\left({N}_D\right)={N}_D=\frac{T}{D}. $$

The proportion of offspring that derives from a particular drone is ${c}_D=\frac{N_D}{T}$.

It follows that ${\sigma}_{c_D}^2=\frac{var\left({N}_D\right)}{T^2}=\frac{1}{TD}$.

Using:

22 $$ {p}_1=D{\sigma}_{c_D}^2+\frac{1}{D}, $$

it follows that:

22a $$ {p}_1=\frac{1}{T}+\frac{1}{D}\approx \frac{1}{D} $$

because *T* is very large.

The average number of drones per drone-producing queen equals $\frac{D}{S}$. This number follows a Poisson distribution such that $var\left({D}_S\right)=\frac{D}{S}$.

We have defined *c*_*S*_ as the proportion of offspring from a particular drone-producing queen, where on average ${c}_S=\frac{1}{S}=\frac{D_S{N}_D}{T}$, using *T* = *SD*_*S*_*N*_*D*_.

With this result,

$$ \begin{array}{l}{\sigma}_{c_S}^2=\frac{var\left({D}_S{N}_D\right)}{T^2}=\frac{1}{T^2}\Big({\overline{N}}_D^2 var\left({D}_S\right)+\\ {}{\overline{D}}_S^2 var\left({N}_D\right)+ var\left({N}_D\right) var\left({D}_S\right)=\\ {}\frac{1}{T^2}\left(\frac{T^2}{D^2}\frac{D}{S}+\frac{D^2}{S^2}\frac{T}{D}+\frac{T}{D}\frac{D}{S}\right)\approx \frac{1}{DS},\end{array} $$

because *T* is very large. The derivation assumes that *D*_*S*_ and *N*_*D*_ are independent. This is a reasonable assumption: the number of offspring of a drone is independent of the number of drones produced by its dam.

Using ${p}_2=S{\sigma}_{c_S}^2+\frac{1}{S}$ (23), it follows that:

23a $$ {p}_2\approx \frac{1}{D}+\frac{1}{S}. $$
